# Unlike conventional methods, extraction of a leadless pacemaker can be performed theoretically and safely by grasping the tines

**DOI:** 10.1016/j.hrcr.2024.05.005

**Published:** 2024-05-14

**Authors:** Masataka Narita, Yoshifumi Ikeda, Kazuhisa Matsumoto, Hitoshi Mori, Kenta Tsutsui, Ritsushi Kato

**Affiliations:** Department of Cardiology, Saitama Medical University International Medical Center, Saitama, Japan

**Keywords:** Leadless pacemaker extraction, Pacemaker failure, Micra, Two single-loop snares, Tricuspid valve, Extraction technique

**Key Teaching Points**

• It is not rare for leadless pacemaker failure to occur after implantation. The treatment strategy for acute-phase complications includes pacemaker extraction.
• The leadless pacemaker extraction is sometimes difficult and involves complications such as pacemaker escape and tricuspid valve injury. The choice of extraction methods can be bothersome.
• We performed an alternative “upside-down” technique to extricate an embedded Micra by directly grasping the tines and retracting it as a safe approach to minimize the risk of injury to the myocardium and tricuspid valve.

## Introduction

The Micra leadless pacemaker (Medtronic, Minneapolis, MN) has a unique structure enabling 4 self-expanding tines to attach to the myocardium of the right ventricle. As yet, there is no genuine removal tool available for Micra extraction. Although a general extraction method is to catch the retrieval head at the opposite side of the tines with a snare, it is difficult to capture this tiny head in a beating heart. Moreover, the exposed tines have the potential to engage with cardiac tissue, raising the risk of myocardial and tricuspid valve injury during retrieval. The innovative technique reported herein, which entailed directly grasping the tines to extract the Micra, offers an alternative resolution to these issues.

## Case report

An 81-year-old woman was referred to another hospital for bradycardia-tachycardia syndrome. When she was leaning against a wall, her legs collapsed, and she hit her head on the floor. She was diagnosed with a traumatic cerebral hemorrhage and hospitalized for further examination and treatment. After admission, the patient returned to sinus rhythm after atrial fibrillation, and a 10-second pause was observed, leading to a diagnosis of bradycardia-tachycardia syndrome. She was transferred to our hospital for pacemaker implantation surgery. Following implantation of the Micra leadless pacemaker, the pacemaker parameters showed an impedance value of 630 Ω and a threshold value of 1.0 V / 0.24 ms. The threshold increased to 2.25 V / 0.24 ms (impedance value 450 Ω) on the fourth day after implantation and 3.63 V / 0.24 ms (impedance value 570 Ω) on the sixth day. The estimated remaining battery life was less than 10 months, thus severely limiting continuous operation. We explained the clinical situation to the patient and her family, who consequently decided on the removal and replacement of the device.

**Figure 1:**
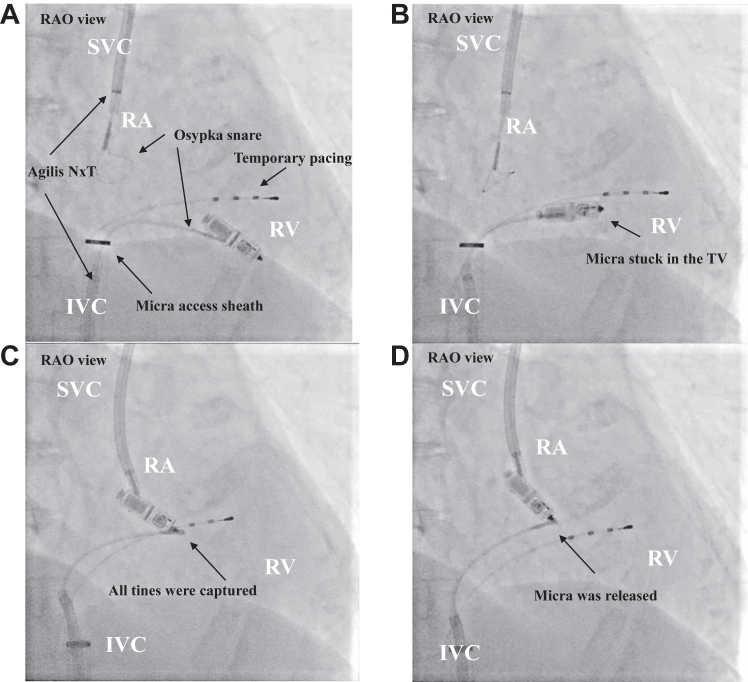
**A:** The Micra pacemaker (Medtronic, Minneapolis, MN) was captured in a single snare via the Micra access sheath and detached from the right ventricle. **B:** The tines of the Micra were stuck in the tricuspid valve and could not be removed intact. **C:** The bottom of the tines were captured directly by the inferior vena cava snare. **D:** The Micra was removed from the tricuspid valve and turned upside down toward the Micra access sheath. IVC = inferior vena cava; RA = right atrium; RAO = right anterior oblique; RV = right ventricle; SVC = superior vena cava; TV = tricuspid valve.

**Figure 2:**
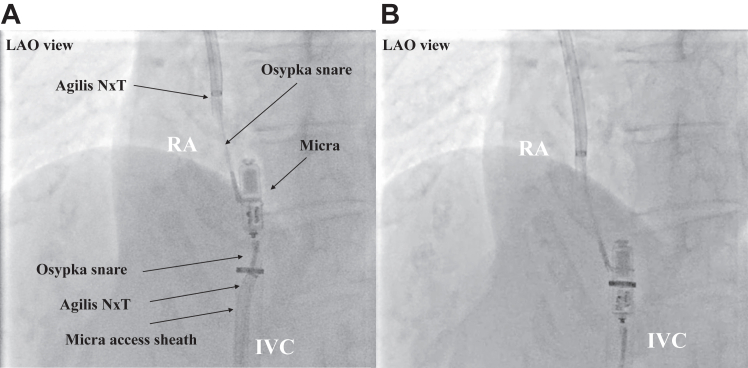
**A, B:** The Micra (Medtronic, Minneapolis, MN) was inserted into the sheath from the tine side and extracted. LAO = left anterior oblique; other abbreviations are the same as in Figure 1.

The 23F Micra access sheath (Medtronic) was inserted into the right femoral vein, and a 5F sheath was placed in the left femoral vein for temporary pacing. Short (61 cm) and long (71 cm) 8.5F steerable sheaths (Agilis NxT; St Jude Medical, St Paul, MN) were inserted via the superior vena cava (SVC) from the right internal jugular vein and into the access sheath via the inferior vena cava (IVC), respectively. We attempted to capture the retrieval head of the Micra with an Osypka snare catheter (Osypka, Rheinfelden-Herten, Germany) from the SVC, but without success. We decided it would be easier to capture the Micra’s body than its retrieval head.^1^ Once the Micra’s body was captured in the Osypka snare with the Agilis NxT via the Micra access sheath, it detached from the right ventricle. However, during this process, the tines became entangled in the tricuspid valve and could not be removed intact (Figure 1A and 1B). The snare from the SVC was then used to grasp the Micra’s body, the bottom of the tines were captured by the IVC snare, and the Micra was released from the tricuspid valve by tying the 4 tines firmly using the IVC snare (Figure 1C and 1D and Supplemental Video 1). When we tried to insert the Micra directly into the Micra access sheath, a few tines became caught on the edge of the entrance to the sheath, interfering with retraction into the sheath. To tackle this problem, the Micra was rotated cautiously to retract it into the sheath (Figure 2 and Supplemental Video 2).

A new Micra was implanted in the apex septum after confirming gooseneck flexion (threshold 0.13 V / 0.24 ms, impedance value 1030 Ω, amplitude 4.6 mV). We confirmed that the pacemaker parameters were normal after surgery, and the patient was transferred to another hospital for rehabilitation.

## Discussion

There were 2 significant findings in this case. First, when extracting a Micra, there is a risk that the exposed tines may hook onto structures such as the myocardium or tricuspid valve, potentially causing tissue damage. Such tines can be released by directly binding them with a snare. Second, the extraction need not necessarily be from the retrieval head side; it can also be retrieved from the tine side (“upside-down” technique).

**Figure 3:**
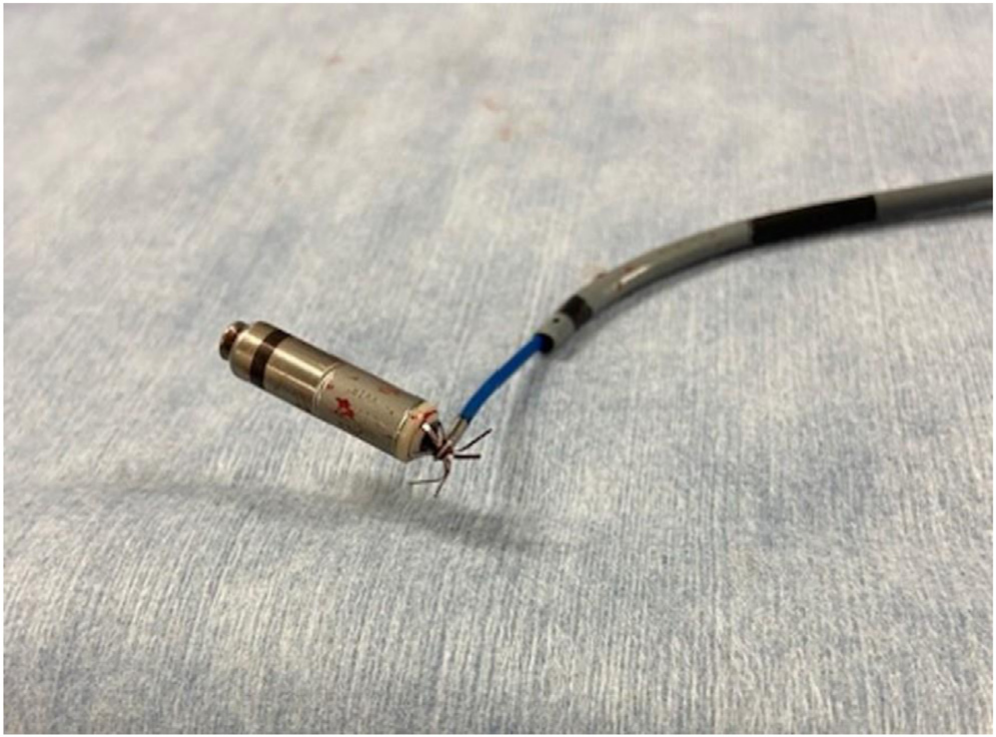
It was shown ex vivo that grasping the base of the tines of the Micra pacemaker (Medtronic, Minneapolis, MN) with a single snare tied the tines.

A dislocated leadless pacemaker may escape into the heart or pulmonary artery.^2^^,^^3^ In some cases, pacemaker extraction may be necessary several weeks after implantation.^1^^,^^4^^,^^5^ An impedance of less than 800 Ω at the time of implantation predicts subsequent threshold elevation.^6^ The impedance of 630 Ω at implantation meets this criterion in this case. While the mechanism of threshold elevation remains unclear, inflammatory changes caused by tines inserted into the myocardium may be a contributing factor. Abrupt threshold elevation is one of the causes of device extraction. The difficulty in removing a leadless pacemaker is the adhesion of the pacemaker to the heart and the FlexFix tines of the Micra becoming entangled in the tissue. The tines may get caught in the tricuspid valve, making it more difficult to remove during Micra extraction. In our case, the tines had become embedded in the tricuspid valve, and forcibly pulling it out may have damaged the valve. Therefore, to capture the bottom of the tines, we used a single-loop snare, which was then able to loosen and release them from the tissue. As we have shown ex vivo, the tines could not be tightened entirely by grasping with a snare; however, they could be retrieved into the sheath while rotating and pulling (Figure 3).

We named this method the “upside-down” technique. This approach is an effective alternative when it is difficult to catch the retrieval head for Micra extraction. It is more difficult to directly catch the head of a Micra that has become completely dislodged and is moving unstably or “dancing.” Tying the tines is relatively simple, as the snare sliding along the Micra body captures the tine side of the device. If we know that it can be retrieved from the tine side, there is little risk of dislodgment when recapturing the retrieval head. Furthermore, this method can be safer because it minimizes the risk of damage to the tricuspid valve and myocardial tissue caused by exposed tines. To enhance the safety of this method, we recommend the concurrent use of the “2-directional snare technique.”^1^ The retention of the tines is less stable than that of the retrieval head, posing a risk of dislodgment. Securing the Micra body with the snare on the opposite side can prevent dislodgment and allow any reattempts.

The critical point is to catch at least 3 tines. In an ex vivo experiment, retraction into the sheath was possible by capturing 3 or 4 tines (Figure 3). Afzal and colleagues^7^ reported that an embolized Micra in a pulmonary artery could be retracted by capturing a single tine. They failed to withdraw it from the tine side into the Micra access sheath because of dislodgment but successfully retrieved it by recapturing the retrieval head.^7^ In the first worldwide report on Micra removal, Karim and colleagues^5^ encountered difficulty extracting the Micra by grasping just 1 tine and needed to use another snare to secure 1 more tine for successful extraction from the tine side.^5^ Our study demonstrates that a single snare is capable of safely extracting the Micra from the tine side. No dedicated retrieval system for the Micra is currently available for rescue from dislodgment. A screw-type leadless pacemaker (AVEIR or Nanostim; Abbott Medical, Abbott Park, IL) has a retrieval catheter system whose safety and efficacy have already been reported.^8^ For extraction of the Micra, it is necessary to use the delivery system and any combination of snares as the retrieval system. Given that the number of leadless pacemaker implantations is expected to increase, operators need to master and refine retrieval techniques. Creating a new technology requires a significant change in thinking. For example, as in this report, changing the structure must be considered to enable “upside-down” capture.

## Conclusion

A Micra leadless pacemaker stuck in the tricuspid valve can be freed by grasping the tines directly, followed by retrieval into the Micra access sheath from the tine side.

## Disclosures

Y.I. received an honorarium from Medtronic Japan as a speaker and a procedure trainer. R.K. received an honorarium from Medtronic Japan as a speaker. All other authors report no conflicts of interest.

## Appendix Supplementary Data

**Supplemental Video 1**. (A) The tines of the Micra were stuck in the tricuspid valve and could not be removed intact. The bottom of the tines were captured directly by the IVC snare. (B)The Micra was removed from the tricuspid valve and turned upside down toward the Micra access sheath. **Supplemental Video 2**. When we tried to insert the Micra directly into the Micra access sheath, a few tines became caught on the edge of the entrance to the sheath, interfering with retrieval into the sheath. The Micra was rotated cautiously and inserted into the sheath from the tine side.
